# Identifying “vital attributes” for assessing disturbance–recovery potential of seafloor communities

**DOI:** 10.1002/ece3.7420

**Published:** 2021-05-04

**Authors:** Rebecca V. Gladstone‐Gallagher, Judi E. Hewitt, Simon F. Thrush, Marco C. Brustolin, Anna Villnäs, Sebastian Valanko, Alf Norkko

**Affiliations:** ^1^ Institute of Marine Science University of Auckland Auckland New Zealand; ^2^ Tvärminne Zoological Station University of Helsinki Hanko Finland; ^3^ Department of Statistics University of Auckland Auckland New Zealand; ^4^ National Institute of Water and Atmospheric Research Hamilton New Zealand; ^5^ Baltic Sea Centre Stockholm University Stockholm Sweden; ^6^ International Council for the Exploration of the Sea (ICES) Copenhagen Denmark

**Keywords:** benthic, beta diversity, cumulative effects, resilience, soft‐sediment, traits

## Abstract

Despite a long history of disturbance–recovery research, we still lack a generalizable understanding of the attributes that drive community recovery potential in seafloor ecosystems. Marine soft‐sediment ecosystems encompass a range of heterogeneity from simple low‐diversity habitats with limited biogenic structure, to species‐rich systems with complex biogenic habitat structure. These differences in biological heterogeneity are a product of natural conditions and disturbance regimes. To search for unifying attributes, we explore whether a set of simple traits can characterize community disturbance–recovery potential using seafloor patch‐disturbance experiments conducted in two different soft‐sediment landscapes. The two landscapes represent two ends of a spectrum of landscape biotic heterogeneity in order to consider multi‐scale disturbance–recovery processes. We consider traits at different levels of biological organization, from the biological traits of individual species, to the traits of species at the landscape scale associated with their occurrence across the landscape and their ability to be dominant. We show that in a biotically heterogeneous landscape (Kawau Bay, New Zealand), seafloor community recovery is stochastic, there is high species turnover, and the landscape‐scale traits are good predictors of recovery. In contrast, in a biotically homogeneous landscape (Baltic Sea), the options for recovery are constrained, the recovery pathway is thus more deterministic and the scale of recovery traits important for determining recovery switches to the individual species biological traits within the disturbed patch. Our results imply that these simple, yet sophisticated, traits can be effectively used to characterize community recovery potential and highlight the role of landscapes in providing resilience to patch‐scale disturbances.

## Graphical abstract

Despite a long history of disturbance–recovery research, we still lack a generalizable understanding of the attributes that drive community recovery potential in seafloor ecosystems. To search for unifying attributes, we explore whether a set of simple traits can characterize community disturbance–recovery potential using seafloor patch‐disturbance experiments conducted in two different landscapes. We consider traits at different levels of biological organization, from the biological traits of individual species within a disturbed patch, to the traits of species at the landscape‐scale associated with their occurrence and numerical dominance across the landscape. Our results imply that these simple, yet sophisticated, traits can be effectively used to characterize recovery potential, but the scale depends on the heterogeneity in landscape biodiversity, highlighting the role of landscap es in providing resilience to patch‐scale disturbances.

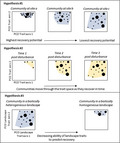

## 1 INTRODUCTION

Ecosystems have a natural ability to recover from disturbance. However, cumulative anthropogenic disturbance increasingly results in localized species loss and landscape homogenization, constraining ecosystem recovery, and shifting the limits of resilience (Devictor et al., 2008; Gámez‐Virués et al., 2015; Hewitt et al., 2010; Hillebrand et al., 2008; Hodapp et al., 2018; Vitousek et al., 1997). There has been a long history of disturbance–recovery research, starting with the pioneering work between the 1910 and 1980s (e.g., Clements, 1916; Connell & Sousa, 1983; Dayton, 1971; Gleason, 1926; Grime, 1974, 1977; Levin & Paine, 1974; MacArthur, 1955; MacArthur & Pianka, 1966; Noble & Slatyer, 1980; Paine & Levin, 1981; Pearson & Rosenberg, 1976; Sousa, 1984), which led to the burgeoning field of disturbance ecology today (reviewed in White & Jentsch, 2001). Nevertheless, we still lack a synthesis of the factors that characterize the recovery potential of ecological communities that can be applied and operationalized in different contexts (Pulsford et al., 2016; White & Jentsch, 2001). Finding generalizable attributes of communities for assessing disturbance–recovery potential is critical as ecosystem management moves toward resilience building decision‐making as a tool to halt the degradation of ecosystem function and services.

The homogenization and loss of species diversity across landscapes (i.e., the biodiversity crisis) reduce ecosystem recovery capacity because it constrains species turnover and the possible options for reassembling communities (Blowes et al., 2019; Hillebrand et al., 2008; Hodapp et al., 2018; de Juan et al., 2013; Mori et al., 2018). Further, as biodiversity is eroded, so is the natural insurance and adaptability that is associated with a level of functional redundancy inherent in many ecosystems (Mori et al., 2013; Oliver et al., 2015). A reduced species pool can thus limit resilience to future changes (because there are less options for reassembling communities), but recovery potential can seem to increase when the reduced species pool is used as the baseline for recovery. Species that remain in simplified homogeneous landscapes are often tolerant environmental generalists that can move around easily and colonize space quickly (Elmgren & Hill, 1997; Pearson & Rosenberg, 1978). While there are many dimensions to community recovery, a better understanding of the role of landscape biotic heterogeneity in governing the recovery trajectory is essential; only with this understanding can we begin to fully grasp the impacts of large‐scale biotic homogenization and the biodiversity crisis on our ecosystems.

Early disturbance–recovery work in terrestrial forest ecosystems derived the concept of “vital attributes” and strategies of plant species that aim to explain ecological succession after forest fires (Grime, 1974, 1977; Noble & Slatyer, 1980). Vital attributes are based on both a species ability to resist a disturbance, and their ability to arrive, establish, and persist at a site following disturbance (Noble & Slatyer, 1980). More recently, “recovery traits” have been used in multiple ecosystem types to characterize recovery dynamics (e.g., Belmar et al., 2019; Carturan et al., 2018; Clarke et al., 2015; Langlands et al., 2011; Villnäs et al., 2018), and these traits usually encompass traits associated with reproductive output and dispersive capacity. These traits and attributes have been quite successful in characterizing terrestrial forest recovery after fires where seedbanks are a dominant source of new recruits and recovery traits tend to focus on the competitive processes that occur during succession (e.g., Clarke et al., 2015; Keith et al., 2007; Ott et al., 2019; Pulsford et al., 2016). However, in many situations (including marine soft‐sediments), recovery traits can be limited as they assume a reliable source population that can supply recruits to recovering patches (Beauchard et al., 2017; Villnäs et al., 2018), an assumption that does not hold true in the context of biotic homogenization and fragmentation across landscapes. That is, traits like dispersive capacity will be unimportant in situations where there is no connected source population to supply the new recruits.

Disturbance–recovery dynamics are heavily influenced by the landscape (e.g., recruit supply and connectivity; de Juan et al., 2013; Pilditch et al., 2015; Saito et al., 2015), local within patch dynamics (e.g., competition and facilitation; Bruno et al., 2003; Dayton, 1971; Norkko et al., 2006; Silliman et al., 2015), and their interactions, which adds complexity and context dependency to community recovery (Leibold et al., 2004; Zajac et al., 1998). Ultimately, multiple scales of space, time, and biological organization are critical (Falk et al., 2019; Gladstone‐Gallagher et al., 2019; Oliver et al., 2015), and the search for vital attributes or recovery traits that are applicable across ecosystem types must consider multiple scales. Adapting concepts of “vital attributes” and “recovery traits” to the soft‐sediments where some species are highly mobile and competition for space is not the key structuring force requires consideration of: (1). the supply of colonists; (2). colonist survivorship; and (3). the ability of colonists to restore ecosystem function (Gladstone‐Gallagher et al., 2019; Levin, 1984; Norkko et al., 2006; Pilditch et al., 2015; Valanko et al., 2015; Villnäs et al., 2018; Whitlatch et al., 1998; Zajac et al., 1998).

We suggest two sets of very simple recovery traits. Our “traits,” broadly termed, consider both the individual species “biological traits” related to life‐history, reproductive, and mobility strategies (“individual recovery traits”), as well as population‐level characteristics that occur at the landscape scale (e.g., species’ occurrence, ability to be dominant and population growth; “landscape‐scale recovery traits”). Individual species traits have been widely used as proxies for recovery and resilience potential (Mori et al., 2013; Piccini et al., 2018; Suding & Goldstein, 2008; Suding et al., 2008; Villnäs et al., 2018). These biological traits indicate how easily the species can colonize and establish in a disturbed patch, but do not discriminate between species that are present locally versus species that are distributed widely across the landscape. However, exploring how species are distributed and positioned in the landscape may provide simple proxies for recovery potential. Species occupancy in the landscape mediates processes such as the supply of new recruits to disturbed areas. Further, in environmentally heterogeneous landscapes, occupancy could also serve as a proxy for the species tolerance to different environmental conditions (Gladstone‐Gallagher et al., 2019; Greenfield et al., 2016).

Here, we test whether these two sets of simple traits that consider both the individual species biological traits and the landscape traits can be used to characterize how soft‐sediment communities recover from disturbance. We draw upon data from the seafloor to test the generality of the traits for predicting recovery in two spatially replicated soft‐sediment disturbance–recovery experiments. Both seafloor contexts have a heterogeneous physical landscape, but they differ in their biotic heterogeneity and regional beta diversity (i.e., the ratio between regional and local species richness) (Whittaker, 1972). One landscape is a subtidal Bay in Northern New Zealand (Kawau Bay) where the benthic macrofaunal community composition across 100 km^2^ is highly heterogeneous (Thrush et al., 2013). Here, a disturbance–recovery experiment was conducted at 8 shallow subtidal soft‐sediment sites (6–12 m depth) in both Austral Autumn and Spring, and recovery of the benthic macrofauna in 4 m^2^ disturbed plots was determined after 5 months. The other landscape is the subtidal seafloor of the Tvärminne‐Hanko Archipelago area in the Baltic Sea (Finland) where the benthic macrofaunal community composition across 50 km^2^ is much less heterogeneous, and benthic biodiversity is naturally restricted due to the brackish‐water environment and the glacial history of the area (Bonsdorff, 2006; Norkko et al., 2010; Villnäs & Norkko, 2011). A disturbance–recovery experiment was established at 15 shallow subtidal soft‐sediment sites (3–6 m depth) in the Northern Hemisphere Summer, and recovery of the benthic macrofauna in 1 m^2^ disturbed plots was monitored at three sampling times during a year.

**FIGURE 1:**
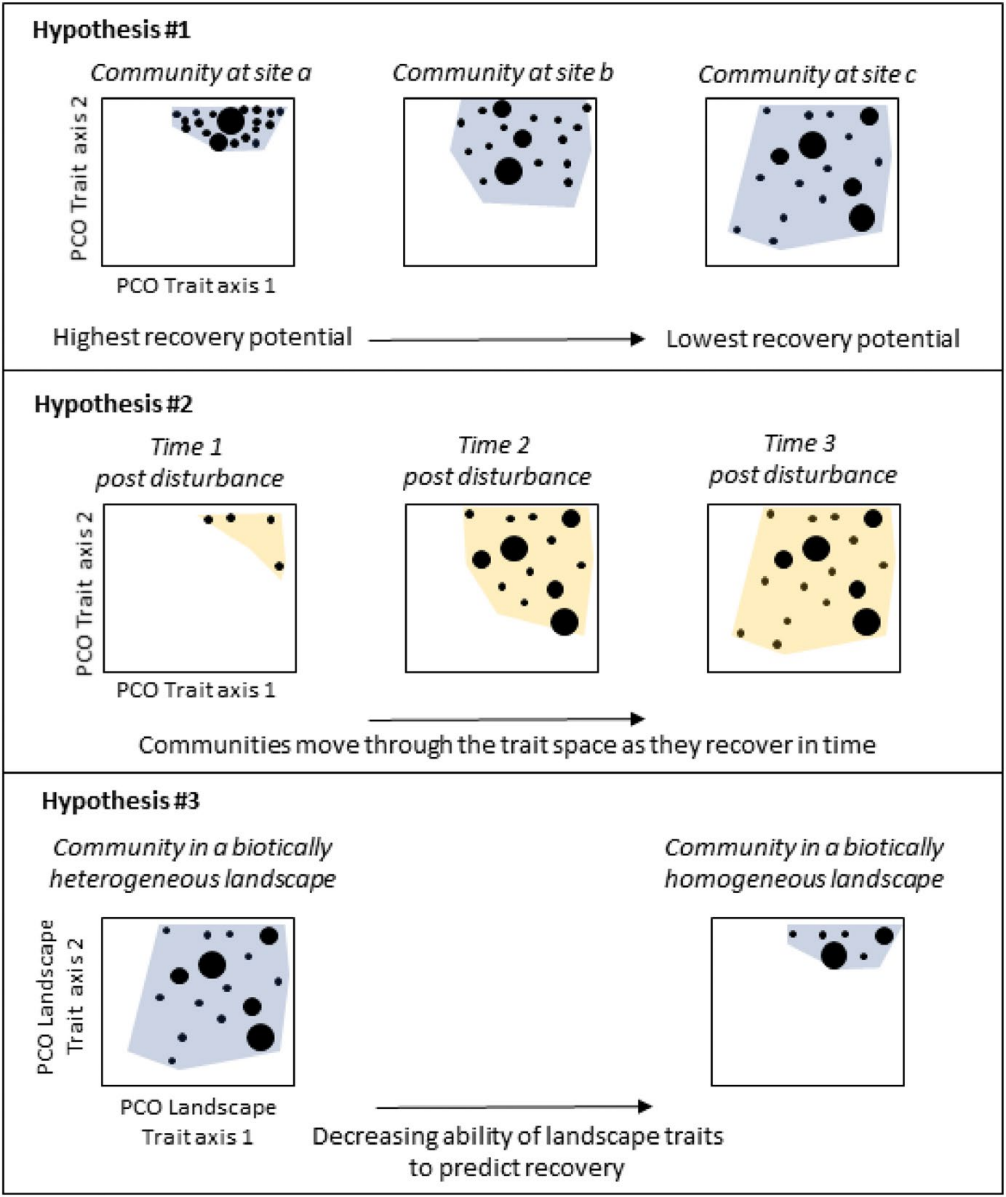
Conceptual diagram illustrating three hypotheses of how recovery traits will relate to community recovery potential in multivariate trait space. For hypothesis #1, species (circles whose size indicate abundance) in three hypothetical communities (a, b, and c) are dispersed/positioned within a multivariate trait space based on their recovery trait composition. For hypothesis #2, the trait composition of species in one hypothetical disturbed community changes through time since the disturbance. For hypothesis #3, species in two hypothetical communities at either end of the spectrum of landscape biotic homogenization are depicted to show how landscape biotic homogenization influences local (within‐site) recovery trait dispersion, which in hypothesis #1 is predicted to influence community recovery potential

These two contrasting datasets encompass different temporal and spatial contexts, but together they represent two ends of a spectrum of biotic landscape homogenization, which allows us to focus on and test the generality of recovery traits for assessing community recovery potential across systems and experiments of different designs. To test the efficacy of our recovery traits in predicting community recovery potential, we use analytical methods that position species and communities in multi‐dimensional ordinations built on their recovery traits (Laliberté & Legendre, 2010; Mouillot et al., 2013), and we expect that the position and dispersion of communities in the multivariate recovery trait space will provide an indication of their potential for recovery from disturbance. We have three hypotheses (see Figure 1 for conceptual diagrams):

1. *Dispersion in recovery trait space is an indicator of community recovery potential*. Conceptually, communities that have high dispersion in the recovery trait space will have a lower recovery potential. High dispersion indicates a wide range of species recovery traits including those species that are the most unique in the trait space (i.e., the rare species), and those which have low mobility and/or infrequent reproduction events. Low dispersion in the trait space indicates communities that comprise species that have similar recovery traits. In most ecosystems (including marine soft‐sediments), it is the rare, large, and long‐lived species that are lost first along a gradient of disturbance (e.g., Hewitt et al., 2010; McGill et al., 2007). Thus, in most cases the contraction of communities in the recovery trait space is likely to result in communities that are resilient and can recover quickly (under current, but not necessarily different, conditions).
2. *Trait composition of recovering communities will change through time*. Communities will move position through time in the trait space as they recover a diversity of recovery traits.
3. *As landscapes become biotically homogenized, the ability of landscape‐scale recovery traits to predict community recovery will decrease*. We expect that landscape recovery traits would be better at predicting recovery in a biotically heterogeneous landscape, while individual traits will be better for predicting recovery in a biotically homogeneous landscape.

## 2 MATERIALS AND METHODS

### 2.1 Datasets

#### 2.1.1 Kawau Bay, New Zealand

**FIGURE 2:**
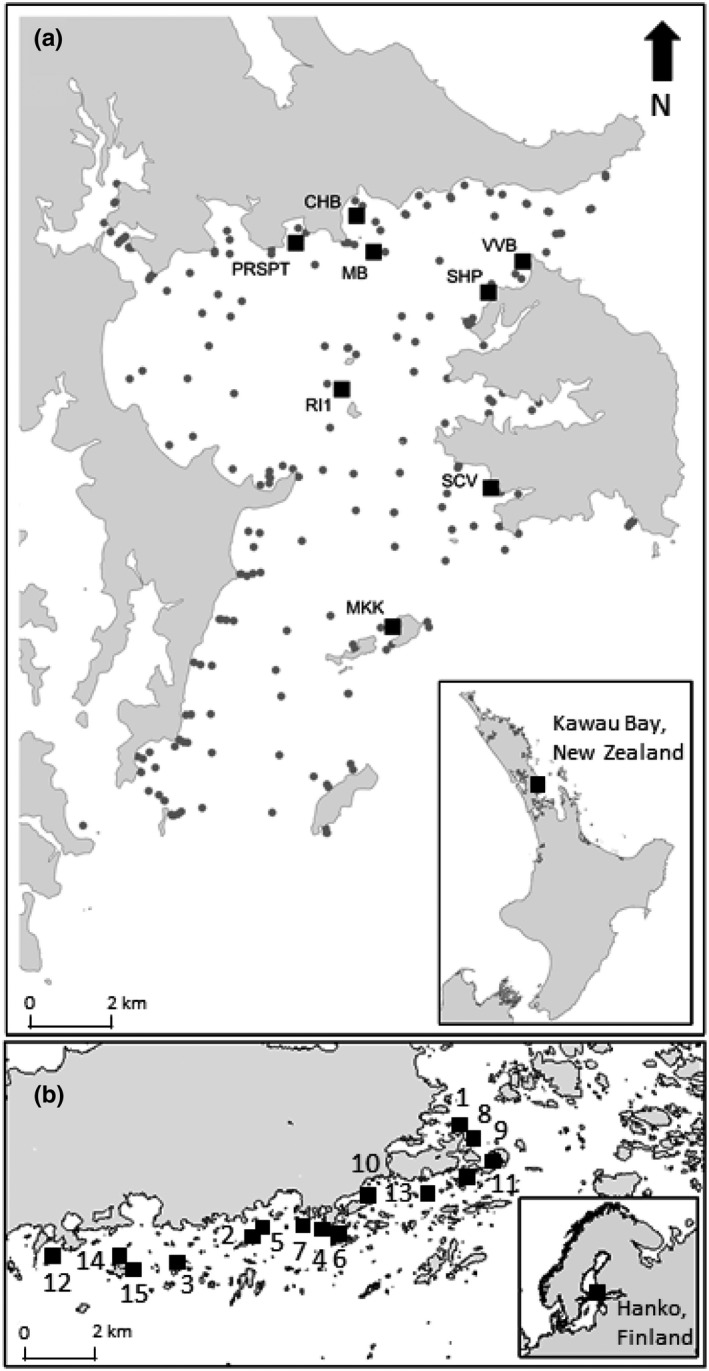
The two study locations: (a) Kawau Bay, New Zealand (modified from Thrush et al., 2013); and (b) Tvärminne‐Hanko Archipelago, Finland, with sites of the disturbance–recovery experiment marked with squares (modified from Valanko, 2012). In A, the grey circles indicate the locations of survey samples

Kawau Bay (hereafter “Kawau”) is in Northern New Zealand and the dataset covers ~100 km^2^ of shallow subtidal area 6–12 m in depth. The regional seafloor diversity and community composition were characterized for Kawau using data from a range of studies dispersed around the bay. At each of the sites marked with grey circles in Figure 2a, the macrofaunal community composition was characterized from these existing datasets to provide context for embedding the experimental work along species diversity and environmental gradients (see Thrush et al., 2013). The regional seafloor taxa richness across Kawau is 334 taxa (measured in a total of 357 samples across sites marked in Figure 2a), and the local within‐site taxa richness ranged between 2 and 40 taxa (mean taxa richness = 14). A disturbance–recovery experiment was conducted at 8 sites within the bay (Figure 2a), chosen to encompass gradients in taxa richness, sediment properties, and physical connectivity to other sites in the coastal landscape (Table 1). At each site, three patches (4 m^2^) were defaunated by randomly placing square sheets of heavy black polythene on the sediment surface, which were weighed down with steel rods to induce anoxia. The plastic sheets were removed after 1 month of deployment, after which time all the macrofauna had been killed by anoxia within the patch. Three undisturbed controls were also marked out at each site (control and disturbed plots were separated by ~3 m).

**TABLE 1:** Mean (±*SE*; *n* = 3) site environmental and biotic characteristics

Site	Depth (m)	Sediment mud content (% <63 µm)	Sediment organic content (%)	Sediment chlorophyll a content (µg/g)	Exposure ranking*	Alpha diversity	Gamma diversity
Tvarminne							27
S6	5.8	18.4 ± 1.9			1	8 ± 0	
S2	5.5	3.0 ± 0.4			2	9 ± 0	
S1	5.1	5.3 ± 0.8			3	9 ± 1	
S3	5.6	6.7 ± 2.0			4	13 ± 1	
S4	6.1	4.9 ± 0.3			5	9 ± 1	
S5	3.7	1.1 ± 0.1			6	8 ± 0	
S11	5.0	0.8 ± 0.1			7	11 ± 0	
S9	4.2	0.3 ± 0.1			8	12 ± 0	
S10	5.2	0.6 ± 0.1			9	11 ± 0	
S8	5.4	0.9 ± 0.1			10	10 ± 0	
S7	4.9	1.5 ± 0.2			11	12 ± 0	
S15	5.7	2.6 ± 1.0			12	11 ± 0	
S14	5.2	0.2 ± 0.1			13	6 ± 1	
S13	4.6	0.1 ± 0.0			14	7 ± 1	
S12	5.4	0.8 ± 0.2			15	8 ± 0	
Kawau							334
MB	10.2	40.09 ± 6.58	3.16 ± 0.22	5.85 ± 0.23	1	13 ± 2	
SCV	9.4	35.47 ± 2.26	3.37 ± 0.54	3.96 ± 0.52	2	7 ± 1	
PRS	7.3	32.32 ± 2.38	3.56 ± 0.22	5.90 ± 0.97	3	17 ± 4	
CHB	6.2	20.05 ± 1.01	2.74 ± 0.05	14.44 ± 2.06	4	10 ± 2	
RI	9.0	17.98 ± 0.37	4.18 ± 0.78	3.55 ± 0.34	5	18 ± 3	
MKK	11.2	13.97 ± 5.37	3.46 ± 0.93	2.64 ± 0.80	6	18 ± 4	
SHP	6.0	8.48 ± 1.79	3.13 ± 0.39	5.68 ± 0.97	7	24 ± 4	
VVB	8.1	2.50 ± 0.88	1.82 ± 0.15	12.04 ± 1.03	8	21 ± 3	

Note

Sites are ordered from lowest to highest physical exposure.

* Since the different studies measured different environmental properties associated with exposure of the sites to physical forcing, we used the available metrics to rank physical exposure of the sites within a region (1 = least exposed). For Tvarminne, we used sediment erosion threshold, grain size, and the amount of deployed gypsum block lost over time in the site exposure ranking. For Kawau, we used sediment mud content as a proxy for exposure.

At Kawau, the patches were left to recover for 5 months after the polythene was removed, after which, two pooled macrofauna cores (10 cm dia. × 10 cm depth) were collected from each control and disturbed plot (Thrush et al., 2013). Core samples were sieved on 500‐µm mesh, macrofauna preserved with 70% isopropyl alcohol, and taxa identified to the lowest possible taxonomic level (usually species). This experiment was repeated twice, and we refer to these two experiments as “phase 1” and “phase 2.” Phase 1 exposed the disturbed plots to colonists in early Austral Autumn, a time when a low supply of larval colonists was expected. Phase 2 initiated colonizations in early Austral Spring, a time with high colonist supply (Thrush et al., 2013). To characterize the different environmental contexts of the 8 sites, two small cores were taken from each plot (2 cm diam. × 2 cm depth) and analyzed for sediment grain size, organic content, and chlorophyll a. Grain size was characterized after hydrogen peroxide digestions and sediment grain sizes were separated by sieves into particle size classes of >2, 2–0.5, 0.5–0.25, and 0.25–0.063 mm. Organic content was determined through weight loss on ignition (400°C for 5 hr) of dried sediment (60°C). Sediment chlorophyll *a* content was determined after pigment extraction using 95% ethanol and then analyzed before and after acidification on a Spectrophotometer. More detailed methods from this experiment can be found in Thrush et al., (2013).

#### 2.1.2 Tvärminne‐Hanko Archipelago, Finland

The Tvärminne‐Hanko Archipelago (hereafter “Tvarminne”) encompasses ~50 km^2^ of shallow subtidal area 4–6 m in depth. Here, a seafloor disturbance–recovery experiment was conducted at 15 sites across a wind‐wave exposure and sediment gradient (Table 1; Figure 2b). The regional seafloor taxa richness across the 15 sites was 27 taxa (measured in 180 samples taken across the 15 sites), and the local within‐site taxa richness ranged between 3 and 17 taxa (mean site taxa richness = 10). At each site, a patch of the seafloor (1 m^2^) was defaunated using the same polythene method as described above for Kawau. An undisturbed control plot at each site was also randomly located 3 m away from the disturbed plot.

Once the polythene was removed (late Northern Hemisphere summer), the patches were left to recover for 5, 35, and 370 days. On each of these days, three macrofauna cores (5.6 cm dia × 10 cm depth) and three sediment grain size cores (2.1 cm dia., 5.0 cm depth) were taken from within each disturbed and control plot. Macrofauna and sediment grain size were analyzed by the same methods as for the Kawau experiment. More detailed methods from this experiment can be found in Valanko (2012) and Norkko et al., (2010). The experiments at both Kawau and Tvarminne mimic complete defaunation from smothering disturbances that generate seafloor anoxia (e.g., from settling algal blooms and sedimentation; Norkko & Bonsdorff, 1996a, 1996b; Thrush et al., 2004).

### 2.2 Analyses

#### 2.2.1 Trait assignment

For all taxa within each dataset, we assigned recovery traits based on two scales of processes. Firstly, taxa were characterized by biological traits that conceptually influence how well they can recover based on their ability to move from the surrounding area and establish themselves and persist in the disturbed patch (hereafter “individual traits”). These individual traits are related to the taxa's life‐history and mobility strategies and are traits that have been implicated in benthic community recovery (Beauchard et al., 2017; Pilditch et al., 2015; Villnäs et al., 2018; Whitlatch et al., 1998). The recovery traits at the individual scale include adult longevity (three trait modalities: <1 year, 1–3 years, or >3 years), larval mobility potential (yes or no), juvenile postsettlement mobility potential (no movement, ability to move in water column, or ability to move in/over the sediment), adult mobility potential (no movement, ability to move in water column, or ability to move in/over the sediment), and maximum size (xs, s, m, or l) (see Table 2 for detailed trait modalities and definitions). While other reproductive traits related to temporal dynamics of recovery such as continuous, seasonal, or single reproductive events could be developed, we did not use them because of the large differences in temporal dynamics linked to the different experiments (boreal and warm temperate), as well as the generally limited of knowledge of these reproductive dynamics for many species. The traits for the New Zealand species were assigned using a biological traits database that was developed by researchers at the National Institute of Water and Atmospheric Research (NIWA) using in house expert knowledge and best available information from the literature (previously described in Rodil et al., 2013). For species and traits that were not available in the NIWA database, and for the Baltic Sea species, online database entries were used including Polytraits (http://polytraits.lifewatchgreece.eu/), WoRMs (http://www.marinespecies.org/), The Arctic Traits (univie.ac.at/arctictraits), and BIOTIC databases (marlin.ac.uk). When trait information could not be found for species, traits at the family level were used and expert opinion filled in the gaps. Fuzzy coding was used to assign traits to species where the assignment of one trait modality was inappropriate (e.g., species that both crawl and swim).

**TABLE 2:** Traits, their modalities, and description of how they conceptually increase community recovery potential

Trait	Modalities	Contribution to community recovery potential
Individual traits		
Presettlement movement potential	Yes larval phase No or minimal larval phase	Organisms with a larval phase have greater potential to colonize disturbed patch
Postsettlement juvenile movement potential	No movement Benthic movement (includes burrowing and crawling) Water column movement (includes swimming, byssus drifting, and rafting)	Organisms degree of mobility dictates how likely it is to colonize disturbed patch as a juvenile
Postsettlement adult movement potential	No movement Benthic movement (includes burrowing and crawling) Water column movement (includes swimming, byssus drifting, and rafting)	Organism degree of mobility dictates how likely it is to colonize disturbed patch as an adult
Maximum size (based on length and body form)	xs (0–10 mm both globulose and streamlined) s (11–20 mm globulose, 11–50 mm streamlined) m (20–50 mm globulose, 50–100 mm streamlined) l (>50 mm globulose, >100 mm streamlined)	Organism size is a proxy for how long it would take for the organism to reach predisturbance population structure in a disturbed patch, where larger bodied individuals are likely to be slower growing and establishing
Adult longevity	Short‐lived (<1 year) Moderate (1–3 years) Long‐lived (>3years)	Organism adult longevity gives an indication of both competitive abilities, as well as how long it influences patch dynamics and other species
Landscape‐scale traits		
Occurrence in the landscape	Rare Moderately rare Moderate Moderately common Common	The occurrence in the landscape provides a proxy for the ability of a species to colonize space and exist across a wide range of environmental conditions (assuming the landscape has high physical heterogeneity)
Ability to be dominant	Low Medium low Medium Medium high High	The maximum abundance of a species across the landscape provides a proxy for the ability of the species to be competitively dominant and therefore able to colonize and establish in a disturbed patch before others
Time to reproductive maturity	Fast maturing (<6 months)	Time to reproductive maturity gives an idea of how quickly a species can establish a population in a disturbed patch after its arrival and recover population numbers. It provides a proxy for population growth.
	Moderate (6 months−1 year)	
	Slow maturing (>1 year)	

Note

Definitions for occurrence and ability to be dominant modalities (ranges within each modality are based on the 25th, 50th, 75th quartiles, and the mean): Occurrence = Rare (occurs in < 2% of samples), Moderately rare (occurs 2%–6% of samples), Moderate (occurs in 6%–9% of samples), Moderately common (occurs in 9%–11% of samples), Common (occurs in > 11% of samples); Ability to be dominant = Low (organism has a maximum abundance of < 2 individuals per core), Medium low (organism has a maximum abundance of 2–5 individuals per core), Medium (organism has a maximum abundance of 6–12 individuals per core), Medium high (organism has a maximum abundance of 13–18 individuals per core), and High (organism has a maximum abundance of >18 individuals per core).

Secondly, taxa were characterized by “landscape‐scale traits,” which consider how population characteristics (numerical dominance and occurrence) influence the taxa's ability to colonize and establish following disturbance. The taxa's “ability to be dominant” and their ability to occur across the landscape (“occurrence”) represent proxies for landscape connectivity and the ability of the taxa to exist across many environmental conditions and these two traits each had 5 modalities. Time to reproductive maturity was also used in the landscape‐scale traits to indicate how fast a taxa can establish and maintain a population in a disturbed patch (3 modalities: <6 months, 6 months‐1 year, or >1 year). While population growth rates will be a function of a number of local drivers, “time to maturity” is a basic trait underlying a species potential population growth rate and was thus used in our study as a proxy for population growth rate in the landscape‐scale traits.

A taxa's “ability to be dominant” was calculated from the maximum abundance of the taxa measured from all samples we had across the landscapes, and the ranges within each trait modality were based on the 25th, 50th, 75th quartiles, and the mean. A taxa's “occurrence” in the landscape was calculated as the percentage of all samples in the landscape that the taxa occurred in, and the ranges assigned to each trait modality were based on the 25th, 50th, 75th quartiles, and the mean. The landscape community context will influence the categorization of these trait modalities. For this reason, we decided to use the Kawau landscape as the baseline, as this is the most diverse community and so the categorization of “occurrence” and “ability to be dominant” modalities were based on the percentiles for the Kawau landscape. At Kawau, occurrences and maximum abundances for each taxon were calculated from the survey samples (Figure 2a) as well as the experimental control plots (*n* = 357). At Tvarminne, occurrences and maximum abundances for each taxon were calculated based on control samples at all sites and times (0, 5, 35, 370 days; *n* = 3 per site per time = 180). “Time to maturity” was assigned using the online trait databases (described above) and expert opinion.

#### 2.2.2 Data analyses

For each dataset, all taxa were positioned in multivariate PCO space (principal coordinates analysis) based on their recovery trait composition (Bray–Curtis dissimilarity between taxa). To demonstrate the importance of traits, we examined trait scores for the first four PCO axes and inspected the trait vector overlays to explore the weighting of the different traits in multivariate space, which determined that none of the traits were unimportant and needed to be removed from the analysis.

To explore how our traits related to recovery (i.e., to test Hypothesis #1; Figure 1), we first calculated the abundance‐weighted multivariate trait dispersion (FDis; i.e., the distance of species trait values from the centre of the trait space (Mouillot et al., 2013)) for each control plot. A community's FDis in either individual‐scale (FDis_I_) or landscape‐scale (FDis_LS_) recovery trait space was then used to explain variability in community recovery among sites within each dataset. We used linear regression models, with recovery as the response variable. Recovery was estimated by calculating Bray–Curtis community similarity between control and disturbed plot communities (i.e., 1‐dissimilarity) (an estimate of recovery back to the original community structure). Prior to calculating Bray–Curtis similarity, taxa abundances were fourth root transformed to down weight the effects of common taxa. The predictor variables in the linear models were the control plot community FDis_I_ and FDis_LS_. We used AIC in backward selection to select which scale of recovery traits (FDis_I_ or FDis_LS_) best explained variation in recovery in each dataset, and we present the best model from this selection (to test Hypothesis #3). A separate linear regression was performed for each temporal sampling. Data were checked for normality and homogeneity of variances.

To aid our interpretation of the linear regressions and to explore Hypothesis #2 (Figure 1), we positioned communities in the PCO ordinations with bubbles overlaid corresponding to the taxa abundances. These ordinations allow visualization of community trait composition. While the first two axes in all cases collectively only explained 41%–53% of the variability, plotting different combinations of the first 3 axes did not alter our interpretation of the results, so only the plots of the first two axes are displayed (see plots in the Appendices). Further, to formally test hypothesis #2, we compared trait composition through time in the Tvarminne disturbed plots (fixed factor with 3 levels: 5, 35, 370 d) using a one‐way PERMANOVA. Trait composition was abundance weighted by multiplying the trait by species abundances in each sample.

As a measure of taxa replacement during recovery, we calculated the proportion of taxa in the disturbed plots that were shared with control plots.

The PERMANOVA was conducted in PRIMER7 with the PERMANOVA+ add on (Anderson et al., 2008). All other analyses were done in R Studio v1.3.959, using the “FD” and “vegan” packages (Laliberté & Legendre, 2010; Oksanen et al., 2019). We present a summary of the key findings in the results section, and we give detailed results and figures in Appendix S1–S2.

## 3 RESULTS

### 3.1 Kawau

**FIGURE 3:**
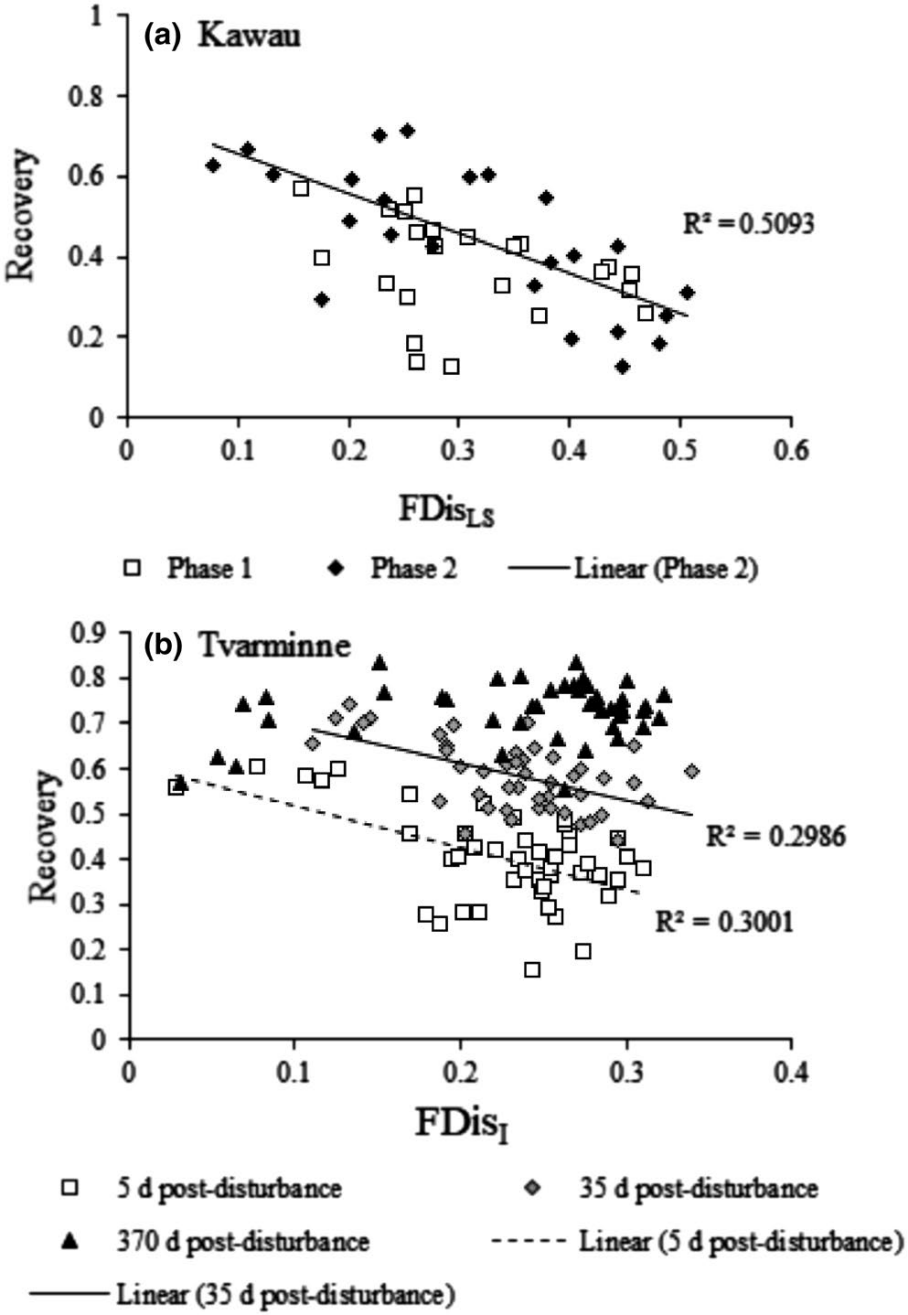
The relationship between recovery and FDis_LS_ at Kawau (a), as well as recovery and FDis_I_ at Tvarminne (b). See Appendix S1 for full linear regression results

**FIGURE 4:**
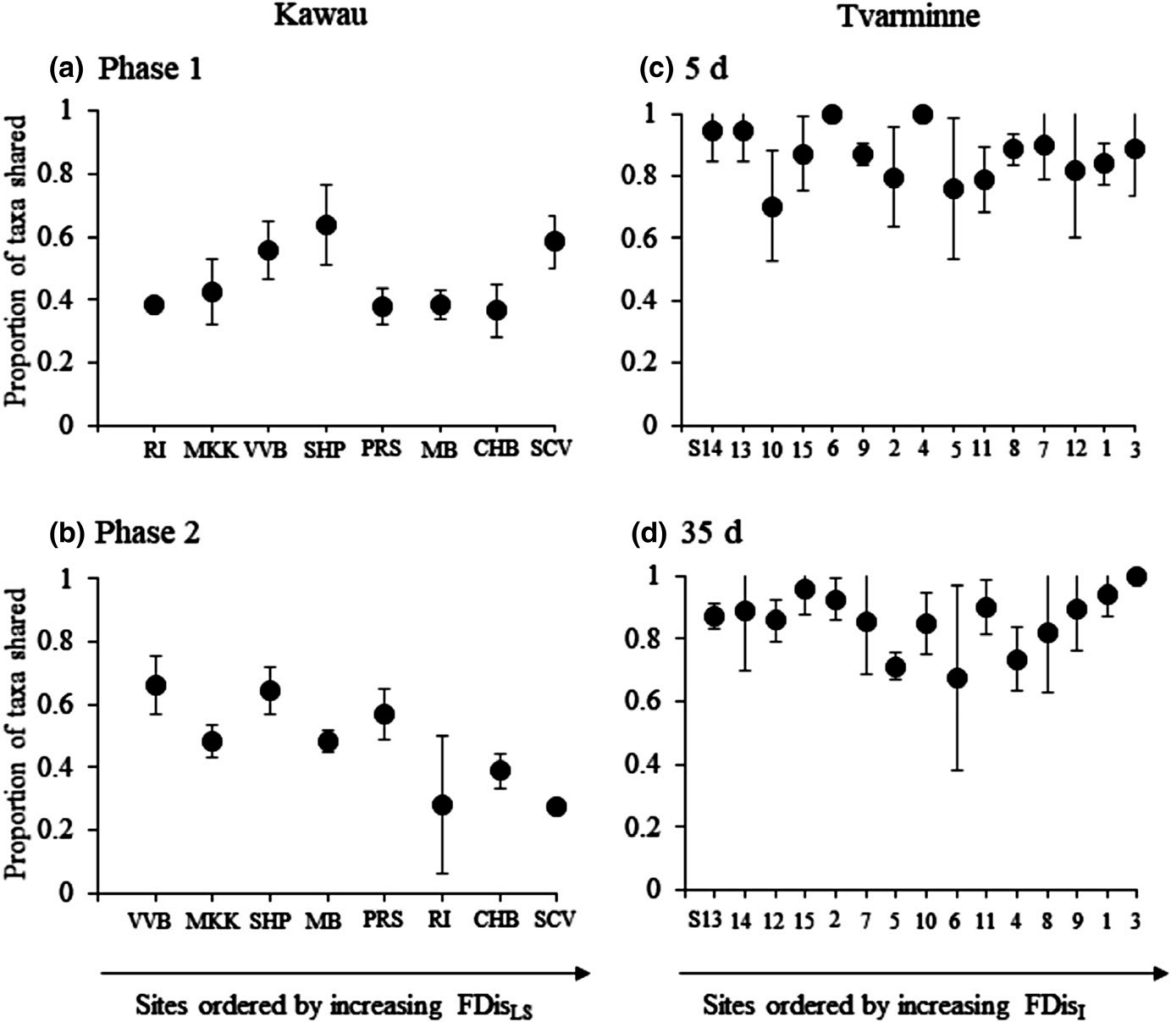
Mean (±*SD*) proportion of the disturbed community taxa that are shared with control communities at Kawau in (a) Phase 1, and (b) Phase 2 and at Tvarminne at (c) 5 days and (d) 35 days post‐disturbance (370 days post‐disturbance is not shown here as the communities were fully recovered by 370 days). At Kawau, sites are ordered from lowest to highest FDis_LS_ (i.e., the best predictor of recovery in this data) in each phase. At Tvarminne, sites are ordered from lowest to highest FDis_I_ (i.e., the best predictor of recovery in this data) at each sampling time

At Kawau, the AIC backward selection removed FDis_I_ from the linear model in both the Phase 1 and Phase 2 experiments indicating that dispersion in individual trait space was not a good predictor of community recovery at Kawau. The resulting linear model that best explained variability in recovery was *recovery ~ FDis_LS_*, which showed a negative relationship between recovery and FDis_LS_ in the phase 2 experiment (black diamonds in Figure 3a), but not in the phase 1 experiment. In this phase 2 experiment, FDis_LS_ explained 51% of the variability in community recovery between sites (linear regression: Phase 1 *R* ^2^ = 0.08, *p* = .2; Phase 2 *R* ^2^ = 0.51, *p = *.00009; full regression results in Appendix S1). The negative relationship between FDis_LS_ and recovery supported Hypothesis #1 that trait dispersion is related to recovery at Kawau. The communities with the highest FDis_LS_ also had the highest taxa replacement during recovery. Communities with lowest FDis_LS_ showed the lowest taxa replacement during recovery (and thus higher recovery and similarity to the undisturbed community composition) (Figure 4b).

### 3.2 Tvarminne

At Tvarminne, the AIC backward selection removed FDis_LS_ from the linear model at 5 days and 35 days post‐disturbance, indicating that a community's dispersion in the landscape‐scale trait space was not a good predictor of community recovery (unlike Kawau). The resulting linear model was *recovery ~ FDis_I_*, which showed a negative relationship between recovery and FDis_I_ at 5 and 35 days postdisturbance (linear regression: 5 days: *R* ^2^ = 0.30, *p* = .0001; 35 days: *R* ^2^ = 0.30, *p = *.0001) (white squares and grey diamonds in Figure 3b). By 370 days, communities were generally fully recovered across the 15 sites (black squares in Figure 3b). The negative relationship between FDis_I_ and recovery supported Hypothesis #1 that trait dispersion was related to recovery at Tvarminne. The trait composition of recovering communities in disturbed plots changed through time supporting Hypothesis #2 (PERMANOVA effect of time: *df* = 2, MS = 31,018, Pseudo‐*F* = 36.5, *p* = .001; all pairwise tests: *p* = .001). At 5 days, communities occupied a relatively small part of the trait space, and the space expanded through time after disturbance (PCO plots show recovering communities in the trait space ordination and can be viewed in Appendix S2). Unlike at Kawau, there was very little species replacement in recovering communities at Tvarminne, and recovery through time followed the trajectory of the reintroduction and increasing abundances of species that were originally in the disturbed patches (Figure 4; Appendix S2).

At Kawau, FDis_LS_ was related to recovery, whereas at Tvarminne, it was FDis_I_ that was related to recovery and this switch supports Hypothesis #3.

## 4 DISCUSSION

Our analysis demonstrates that some very simple traits can be effective at predicting recovery, and the comparison of the importance of species and landscape traits in the two experiments reveals interesting insights into the spectrum of different recovery outcomes. Multivariate trait dispersion was important in both locations (Hypothesis #1), but the mechanism of recovery and the scale of recovery traits differed. In Kawau, multivariate trait dispersion related to the level of species replacement in recovering communities (the mechanism for recovery). In Tvarminne, the mechanism of recovery was the gradual reintroduction of species to the original community composition (rather than species replacement). In this biotically homogeneous context (Tvarminne), individual‐scale recovery traits were related to the speed of this species reintroduction, and the trait composition of recovering communities changed through time since disturbance (supporting Hypothesis #2). However, in a biotically heterogeneous context (Kawau), it was the landscape‐scale traits that best predicted recovery (Hypothesis #3).

In a species rich and heterogeneous landscape (Kawau), the individual recovery traits do not provide a good assessment of community recovery potential, and instead, our results emphasize the key role of the regional landscape diversity in driving recovery (corroborating previous findings in this ecosystem Thrush et al., 2008, 2013). Local within‐site communities with high dispersion in the landscape trait space showed the highest level of species replacement during recovery (and therefore “lowest recovery” back to the original community structure; Figure 4b). These communities conceptually contain more species with lower recovery potential, so during recovery, the species that cannot recover are replaced by new species supplied by the landscape. Thus, recovery displayed an element of “randomness” associated with species replacement. Stochasticity signals that in a biotically heterogeneous landscape, there are more possible options for recovery (than the simple reintroduction of the original species), which increases the likelihood of priority effects and a diverse array of successional pathways (which could also include hysteresis pathways) (Gleason, 1926).

High biodiversity in the landscape opens more options for recovering communities. However, different recovery outcomes have been found to rely on the movement of species and the timing of disturbance with respect to the timing of reproduction and recruitment (e.g., Levin, 1984; Thrush et al., 2013). In Kawau, the landscape‐scale recovery traits were important for explaining community recovery potential in Austral spring when there are high abundances of colonists (Phase 2 experiment), but not in Austral autumn when colonist supply was lowest (Phase 1 experiments). This finding corroborates the efficacy of our landscape‐scale traits as proxies for the ability of species within the landscape to supply recruits to the patch scale. Traditionally, assessments of the ability of the landscape to supply recruits to recovering patches have relied on hydrodynamic dispersal models that require a good understanding of site‐specific hydrodynamic processes and physiological and behavioral characteristics of larvae (e.g., Lundquist et al., 2004). These models often also assume that larvae that arrive in the patch will be able to establish. Whereas occupancy considers both the potential connectivity across the landscape and in environmentally heterogeneous landscapes, it also tells us how likely a species is to be able to exist in a wide range of environmental conditions. While occupancy is simple to calculate, when coupled with estimates of a species time to maturity, our results suggest it could provide an alternative proxy for dispersal and connectivity when the data demands of hydrodynamic dispersal models are not met.

Since the Baltic seafloor is a naturally low‐diversity system, it might be envisioned as a proxy for a future world, where current trajectories of biodiversity loss predict increases in biotic homogenization and fragmentation across landscapes (Blowes et al., 2019; Brustolin et al., 2019). In this low‐diversity ecosystem, recovery was very different to the stochastic potential that describes the recovery at Kawau. Recovering communities at Tvarminne showed a gradual, and more deterministic, reintroduction of species back into the disturbed patches through time and this recovery trajectory was relatively similar across the sites. There was no species replacement in recovering communities, and the recovery could be easily visualized based on the individual‐scale recovery traits (Appendix S2). Here, dispersion in the recovery trait space (FDis_I_) was related to variability in initial recovery among sites, where high FDis_I_ was related to slower initial recovery. This constrained number of outcomes likely decreases the recovery and adaptive potential of this system in the long term and in the face of disturbances that alter the environmental conditions (e.g., climate change). Even though the two experiments measured recovery at different times after disturbance, they confirm that recovery back to the original species composition is relatively fast in a species poor system (i.e., recovery was 44%–74% after 35d at Tvarminne compared with 13%–71% recovery after 5 months at Kawau). While homogeneous communities are rapidly recovered, there is no potential enhancement of biodiversity with disturbance (instead disturbance results in abundance changes). This biotically homogenous system could be considered a stable state that recovers fast but may be vulnerable to future environmental changes.

Our analysis focussed on a divergence in drivers of community recovery, and however, there are also implications for the recovery of ecosystem function. Species replacement was low in recovering communities at Tvarminne, indicating that community recovery could be linked to the recovery of the original ecosystem functions, but homogenous biotic landscapes and low species richness can also imply limited multifunctionality (Villnäs et al., 2018), and functional recovery may take considerable time with high disturbance frequency impeding development of pre‐disturbance functions (Norkko et al., 2013). However, the implications for Kawau are more difficult to assess, because variation in species distributions across landscapes and variation in community composition can influence functional performance and multifunctionality (Schenone & Thrush, 2020; Siwicka & Thrush, 2020). Thus, new research investigating how these recovery traits are tied to traits that are linked to multiple ecosystem functions represents a new research focus for ecosystem dynamics.

Our assignment of recovery traits to communities recovering from experimental disturbance has helped in operationalizing some of the theory on successional recovery processes in marine soft‐sediments (e.g., Pearson & Rosenberg, 1976). Recovering communities at Tvarminne expanded in the individual recovery trait space through time since disturbance (hypothesis #2), and this pattern was generally similar across the 15 sites (Appendix 2). This type of analysis is a tool that could be used to assess the recovery potential of different communities to disturbance. Ecological risk assessments could use this visual tool to position communities in recovery trait space and characterize communities (or species) as having either high or low recovery potential based on their trait composition. Further, a community's FDis appears to be a good proxy for the speed of recovery following disturbance. When coupled with the species contribution to functions and services, this could provide insightful information about the priorities for protection and the efficacy of restoration and conservation efforts (where sites that are likely to be important for recovery can be targeted by restoration efforts). At Tvarminne, the patterns were deterministic and consistent across sites with different environmental characteristics signaling that there is some generality in this indicator of recovery potential in species poor systems (Appendix S2).

The ability of benthic ecologists to draw generalizations from soft‐sediment disturbance–recovery experiments has been limited by the lack of consistent experiments and the complexity of recovery processes that result in a potentially wide range of possible recovery “outcomes.” Our contribution builds on the extensive literature identifying an array of multi‐scale factors that drive soft‐sediment recovery dynamics and focused on the two ends of a spectrum of seascape regional diversity to test hypotheses about how traits at different scales of biological organization are implicated in disturbance–recovery dynamics. There is a need for ecological generalizations to be developed from experiments and our analysis of these very different benthic systems could be considered a starting point in this process, as it bookends the spectrum of landscape versus local processes in driving recovery. There is now a need to test where along the spectrum of seascape biotic heterogeneity the regional species pool begins to limit recovery. This information is essential to understand how the resilience of small conservation areas is impacted by changing regional species diversity.

It is critical that we continue the search for generalities that can inform how we assess vulnerability of communities to disturbance and stress. We focused on identifying some simple traits that can inform our ability to assess community recovery potential in a range of contexts and we liken this to the search for “vital attributes” that have historically been used to describe the persistence of plant communities through disturbance (Noble & Slatyer, 1980). Our positioning of recovering communities in multivariate recovery trait space enables growth of a more sophisticated understanding of recovery potential than the traditional assignment of species as *r* or *k* strategists. We show that in biotically heterogeneous landscapes the vital attributes should be characterized by the landscape‐scale traits (i.e., species occurrence, ability to be dominant, and speed of maturity). However, as landscapes become biotically homogenized, these landscape traits have lesser importance, but some individual species biological traits can be used to help assess community recovery potential. As communities become homogenized, the opportunities for recovery become constrained (due to less regional species richness) and deterministic (i.e., responses are less stochastic in terms of species turnover). Low stochasticity is likely linked with lower community recovery potential, so while we strive for more generalizable traits that can predict recovery, we must also be able to accept that an element of “randomness” in community recovery trajectories probably provides higher resilience and an increased ability to recover from a wider range of disturbances simply due to chance.

## CONFLICTS OF INTEREST

The authors declare no conflicts of interest.

## AUTHOR CONTRIBUTION

**Rebecca Gladstone‐Gallagher:** Conceptualization (lead); Formal analysis (lead); Investigation (lead); Methodology (equal); Writing‐original draft (lead); Writing‐review & editing (lead). **Judi Hewitt:** Conceptualization (equal); Data curation (equal); Formal analysis (equal); Methodology (equal); Writing‐review & editing (equal). **Simon Thrush:** Conceptualization (equal); Data curation (equal); Methodology (equal); Writing‐review & editing (equal). **Marco C. Brustolin:** Conceptualization (equal); Formal analysis (equal); Methodology (equal); Writing‐review & editing (equal). **Anna Villnäs:** Conceptualization (equal); Data curation (equal); Writing‐review & editing (equal). **Sebastian Valanko:** Data curation (equal); Methodology (equal); Writing‐review & editing (equal). **Alf Norkko:** Conceptualization (equal); Data curation (equal); Methodology (equal); Writing‐review & editing (equal).

## Supporting information

Supplementary Material

Supplementary Material

Supplementary Material

Supplementary Material

Supplementary Material

## Data Availability

Data are available from the Dryad Digital Repository at https://doi.org/10.5061/dryad.msbcc2fxk.
