# Supplementary figures and images for: Identifying “vital attributes” for assessing disturbance–recovery potential of seafloor communities
Source: Ecol Evol. 2021 May 4;11(11):6091–103. doi: 10.1002/ece3.7420 (PMC8207434; doi:10.1002/ece3.7420)

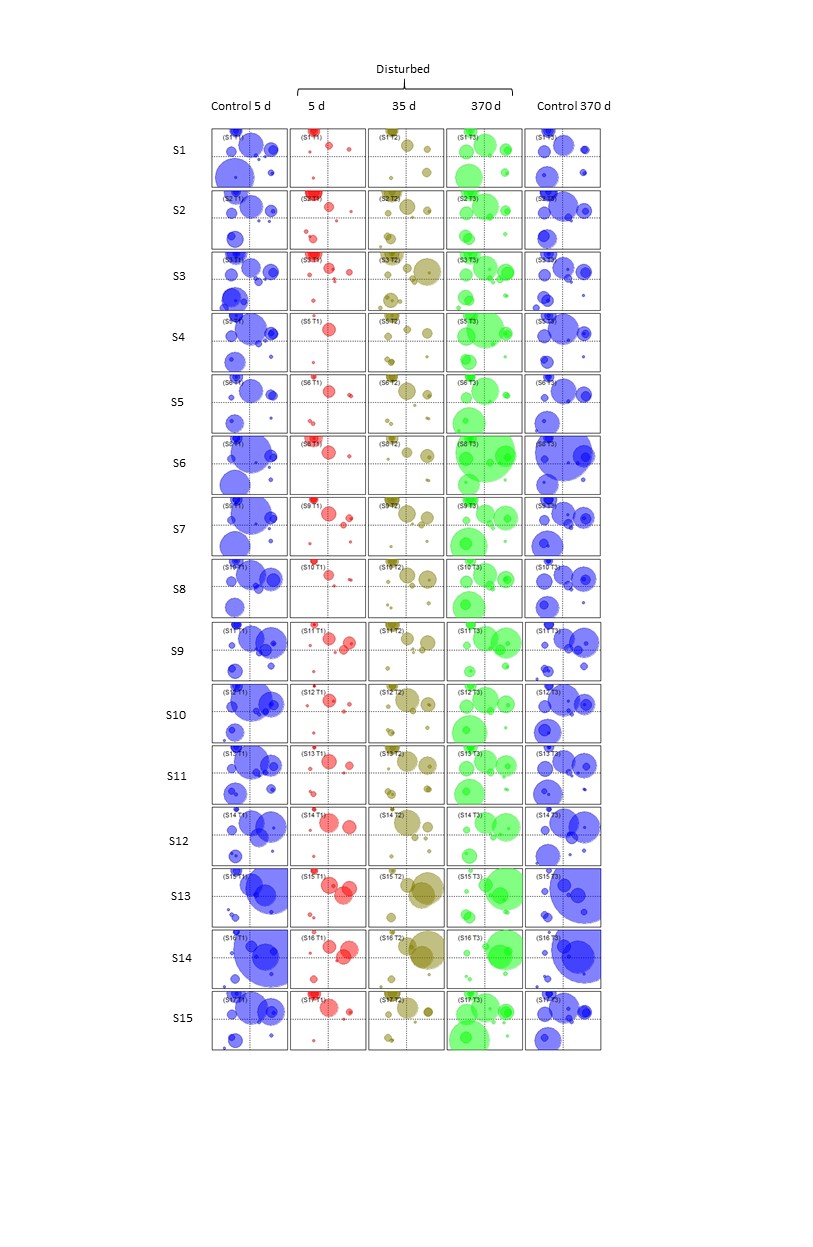

Supplement: Supplementary file 1 — Supplementary Material [file ECE3-11-6091-s002.jpg]

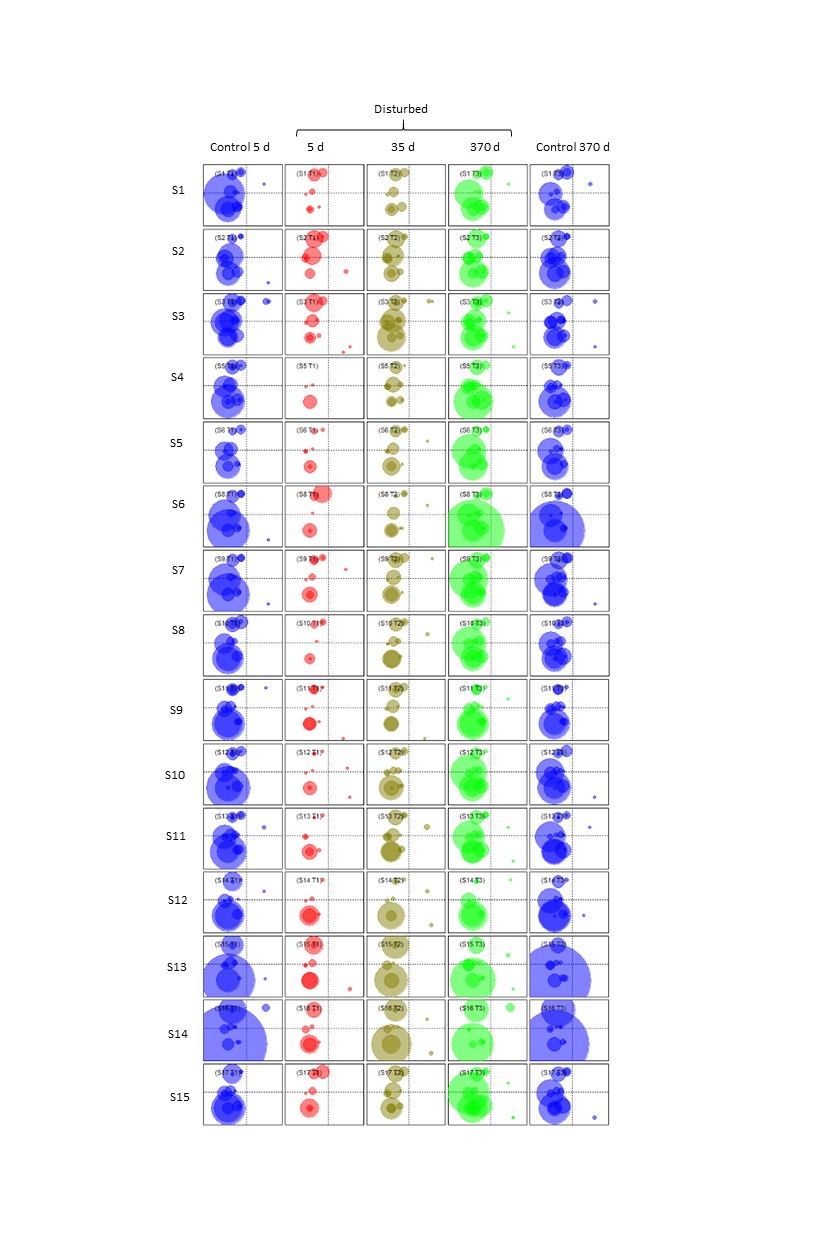

Supplement: Supplementary file 2 — Supplementary Material [file ECE3-11-6091-s001.jpg]

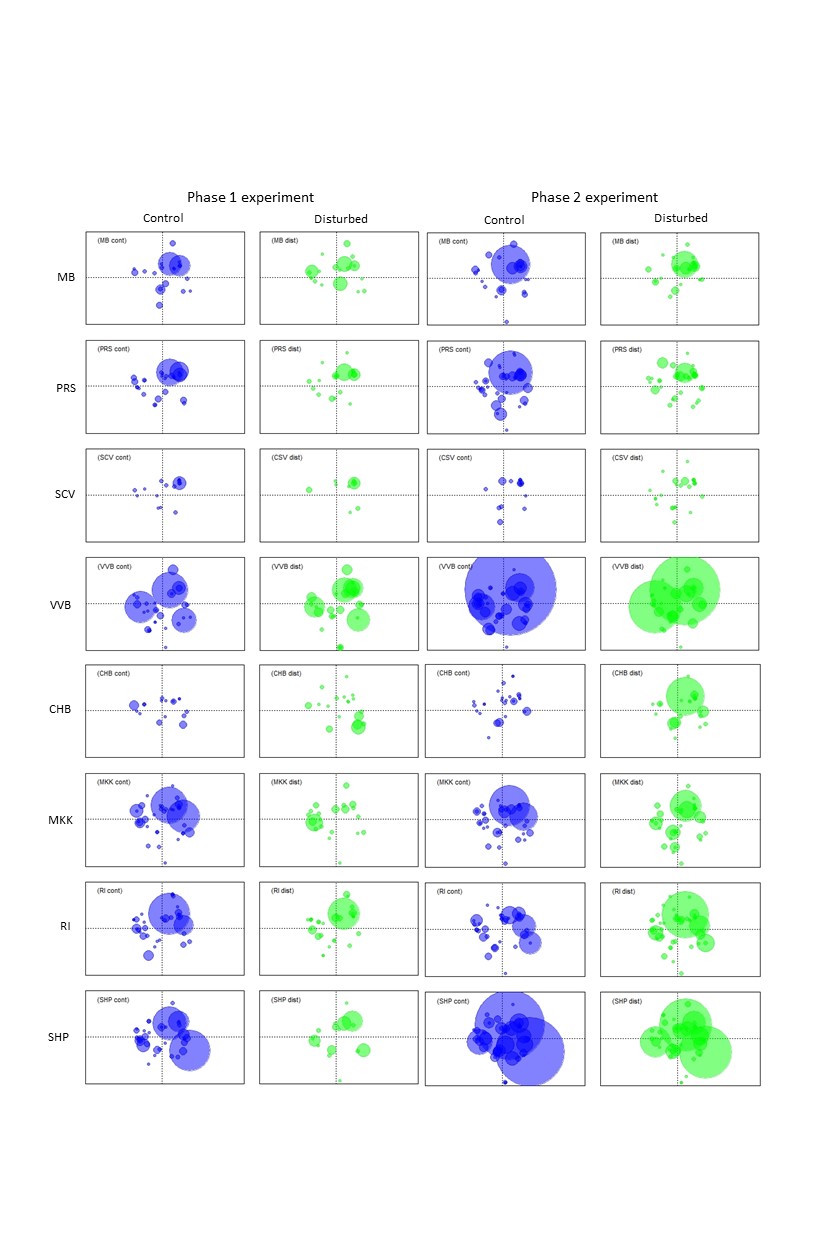

Supplement: Supplementary file 3 — Supplementary Material [file ECE3-11-6091-s003.jpg]

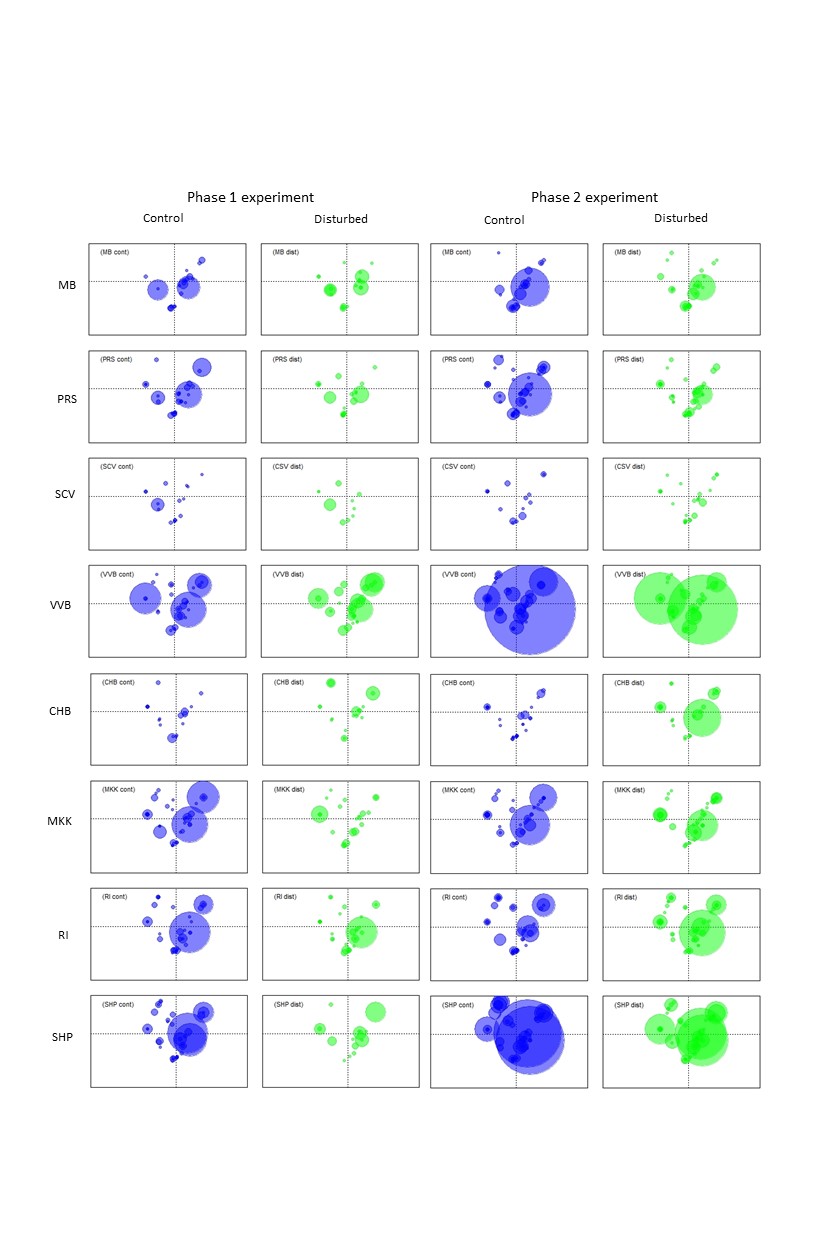

Supplement: Supplementary file 4 — Supplementary Material [file ECE3-11-6091-s005.jpg]
