# Supplementary material for: Identifying “vital attributes” for assessing disturbance–recovery potential of seafloor communities
Source: Ecol Evol. 2021 May 4;11(11):6091–103. doi: 10.1002/ece3.7420 (PMC8207434; doi:10.1002/ece3.7420)
Supplement: Supplementary file 5 — Supplementary Material [file ECE3-11-6091-s004.docx]

# Appendix 1: Linear regression results

**Table A1.1.** Linear regression of the relationship between recovery (response variable) and control plot community dispersion in trait space (FDis_I_ and FDis_LS_; predictor variables). The models presented here are the best models that were selected based on backward selection of AIC.

| **Relationship** | **Estimate** | **SE** | **t-value** | **Pr (>\|t\|)** |  |
| --- | --- | --- | --- | --- | --- |
| Tvarminne 5 days |  |  |  |  |  |
| *Recovery ~ FDis_I_* |  |  |  |  | Residual SE (43 df) = 0.08701, R^2^ = 0.3001, F-statistic (1 and 43 df) = 18.43, *p* = 0.0001 |
| Intercept | 0.61328 | 0.05078 | 12.077 | <0.00001 |  |
| FDis_I_ | -0.93411 | 0.21756 | -4.294 | 0.0001 |  |
| Tvarminne 35 days |  |  |  |  |  |
| *Recovery ~ FDis_I_* |  |  |  |  | Residual SE (43 df) = 0.06472, R^2^ = 0.2986, F-statistic (1 and 43 df) = 18.3, *p* = 0.0001 |
| Intercept | 0.77614 | 0.04575 | 16.967 | <0.00001 |  |
| FDis_I_ | -0.81999 | 0.19167 | -4.278 | 0.0001 |  |
| Tvarminne 370 days |  |  |  |  |  |
| *Recovery ~ FDis_LS_* |  |  |  |  | Residual SE (43 df) = 0.05785, R^2^ = 0.1842, F-statistic (1 and 43 df) = 9.708, *p* = 0.003 |
| Intercept | 0.6545 | 0.0252 | 25.976 | <0.00001 |  |
| FDis_LS_ | 0.3981 | 0.1278 | 3.116 | 0.003 |  |
| Kawau Phase 1 |  |  |  |  |  |
| *Recovery ~ FDis_LS_* |  |  |  |  | Residual SE (21 df) = 0.1209, R^2^ = 0.08887, F-statistic (1 and 21 df) = 2.048, *p* = 0.2 |
| Intercept | 0.50002 | 0.09298 | 5.378 | 0.00003 |  |
| FDis_LS_ | -0.41140 | 0.28745 | -1.437 | 0.2 |  |
| Kawau Phase 2 |  |  |  |  |  |
| *Recovery ~ FDis_LS_* |  |  |  |  | Residual SE (22 df) = 0.1269, R^2^ = 0.5093, F-statistic (1 and 22 df) = 22.83, *p* = 0.00009 |
| Intercept | 0.75564 | 0.06986 | 10.817 | <0.00001 |  |
| FDis_LS_ | -0.99153 | 0.20752 | -4.778 | 0.00009 |  |

# Appendix 2: PCOs showing the recovering communities’ trait composition in the multivariate recovery trait space.

**Tvarminne - individual recovery trait space**

At Tvarminne, the first two PCO axes collectively explained 53% of the variability in individual traits (axes 3 and 4 not shown here explained 16 and 13%, respectively). At all sites, the disturbed communities were initially (i.e. after 5 days) largely reduced to the top panels of the PCO trait space (Fig. A2.1). This part of the ordination is considered to have taxa with the highest recovery potential. As the recovery trajectory progressed (35 and 370 days) the community began to spread to the bottom panels of the ordination. The bottom panels of the ordination contain the taxa that are expected to have the lowest recovery potential. After 370 days the community spread in the disturbed plots closely resembled that of control plots, indicating close to full recovery (Fig. A2.1). Visually, the movement of recovering communities in the individual trait space through time was similar across the 15 sites, indicating generality.

**Tvarminne – landscape-scale recovery trait space**

The ordination of taxa at Tvarminne based on landscape-scale traits explained 49% of the variability in the first two axes (axes 3 and 4 not shown here explained 15 and 13%, respectively). Visually, recovering communities moved in the PCO through time, where initially (5 days) the community occupied a small area of the multivariate trait space and as recovery progressed the community expanded into other parts of the space (Fig. A2.2). After 370 days, communities in both disturbed and control plots occupied similar multivariate trait spaces.


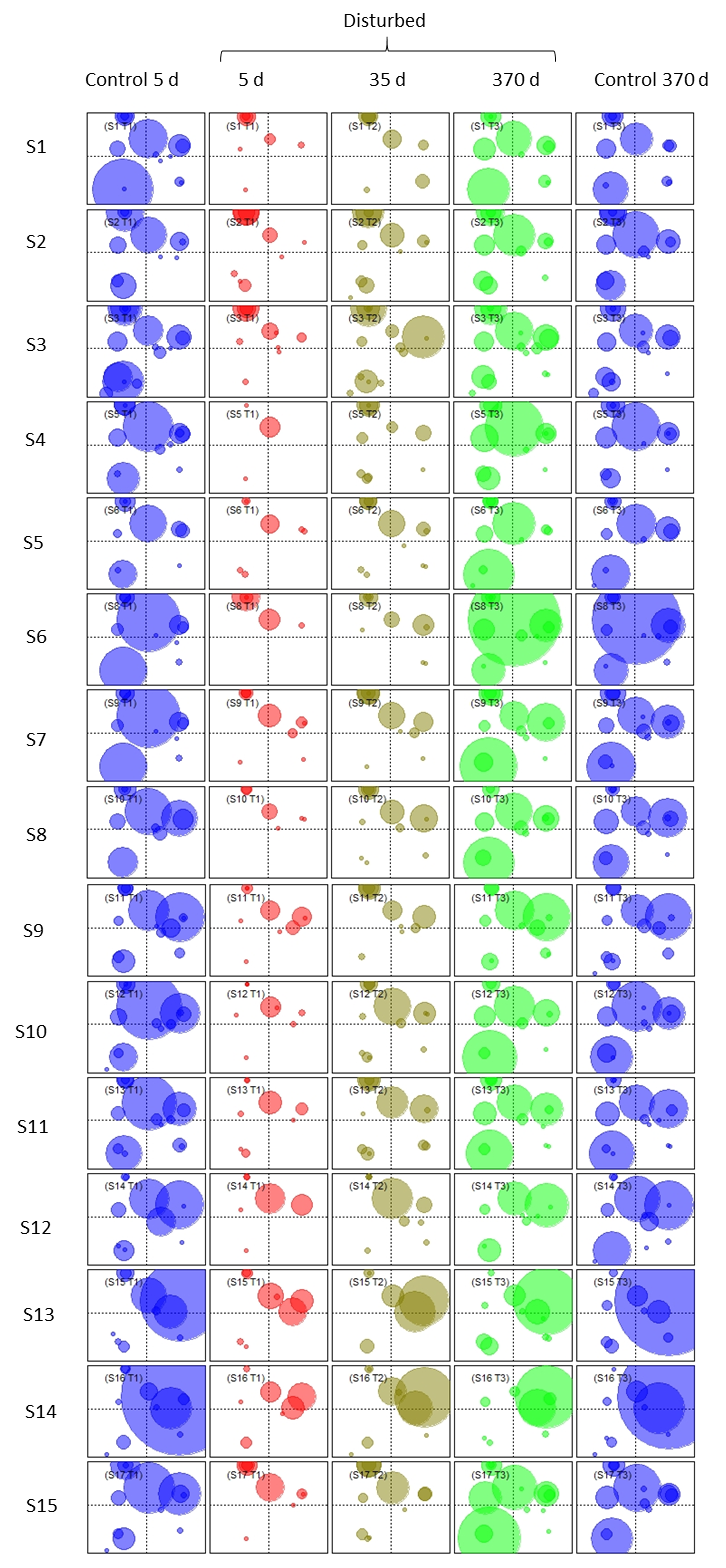


**Fig. A2.1**: PCO ordinations of the community at each site for controls (at 5 and 370 days; blue bubbles) and then disturbed plots through time (5-370 days; red, yellow, green bubbles) at Tvarminne. The plots show the taxa position in the individual recovery trait space on axis 1 and 2, and the size of the bubble indicates the abundance of the taxa (sum of three cores in the plot).

**
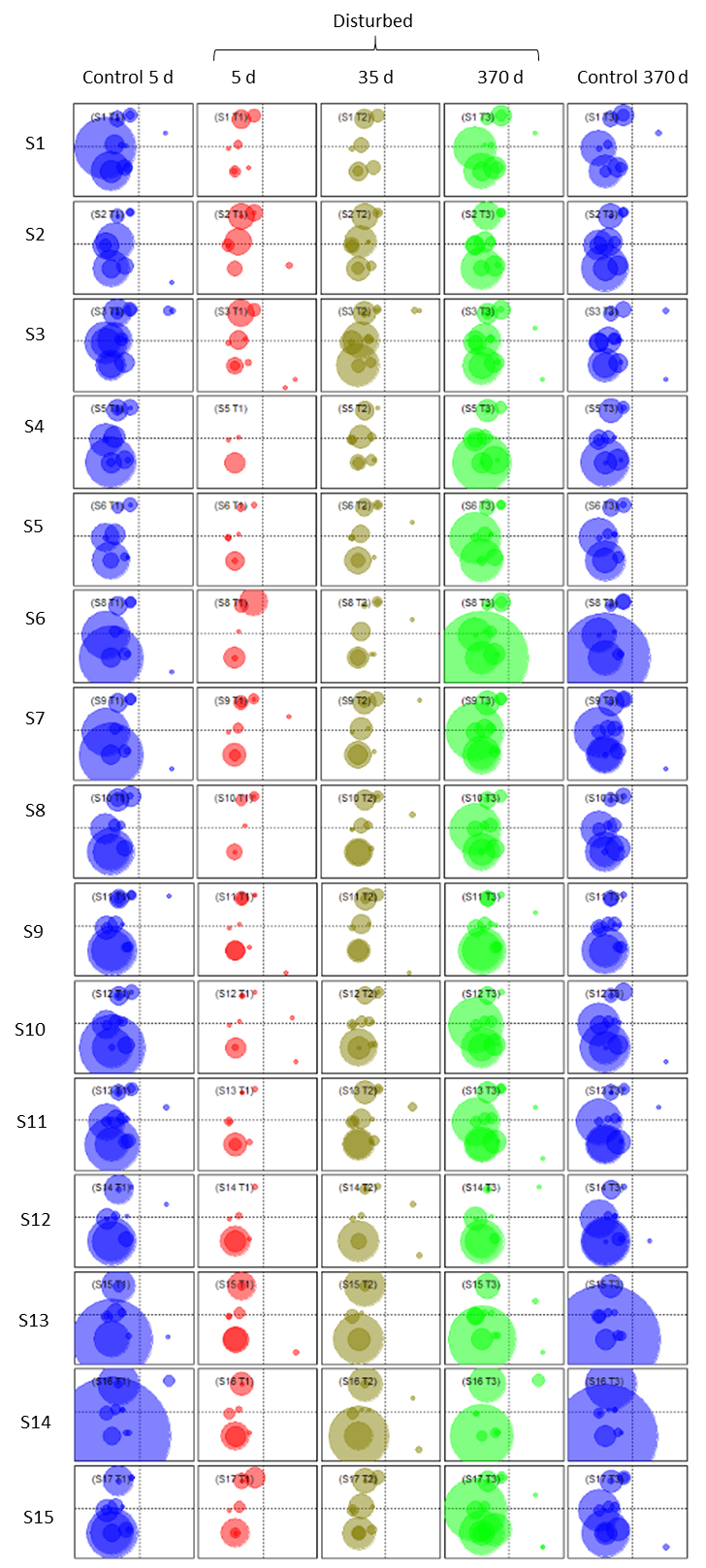
**

**Fig. A2.2**: PCO ordinations of the community at each site for controls (at 5 and 370 days; blue bubbles) and then disturbed plots through time (5-370 days; red, yellow and green bubbles) at Tvarminne. The plots show the taxa position in the landscape recovery trait space on axis 1 and 2, and the size of the bubble indicates the abundance of the taxa (sum of three cores in the plot).

**Kawau – individual recovery trait space**

The first two axes of the PCO explained 44% of the variability in individual traits at Kawau (axes 3 and 4 not shown here explained 18 and 12%, respectively; Fig. A2.3). There were no obvious visual patterns and control and disturbed communities occupied similar parts of the trait space. However, these ordinations indicated that there was a high degree of taxa replacement in the disturbed communities.

**Kawau – landscape-scale recovery trait space**

At Kawau, the PCO ordination of taxa based on their landscape-scale traits explained 41% of the variability in the first two axes (axes 3 and 4 not shown here explained 17 and 10%, respectively; Fig. 3B). When positioning the control and recovering communities in this landscape-scale trait multivariate space (Fig. A2.4), there were no obvious visual patterns. However, the ordinations indicate that there is a high degree of taxa replacement in the disturbed communities.


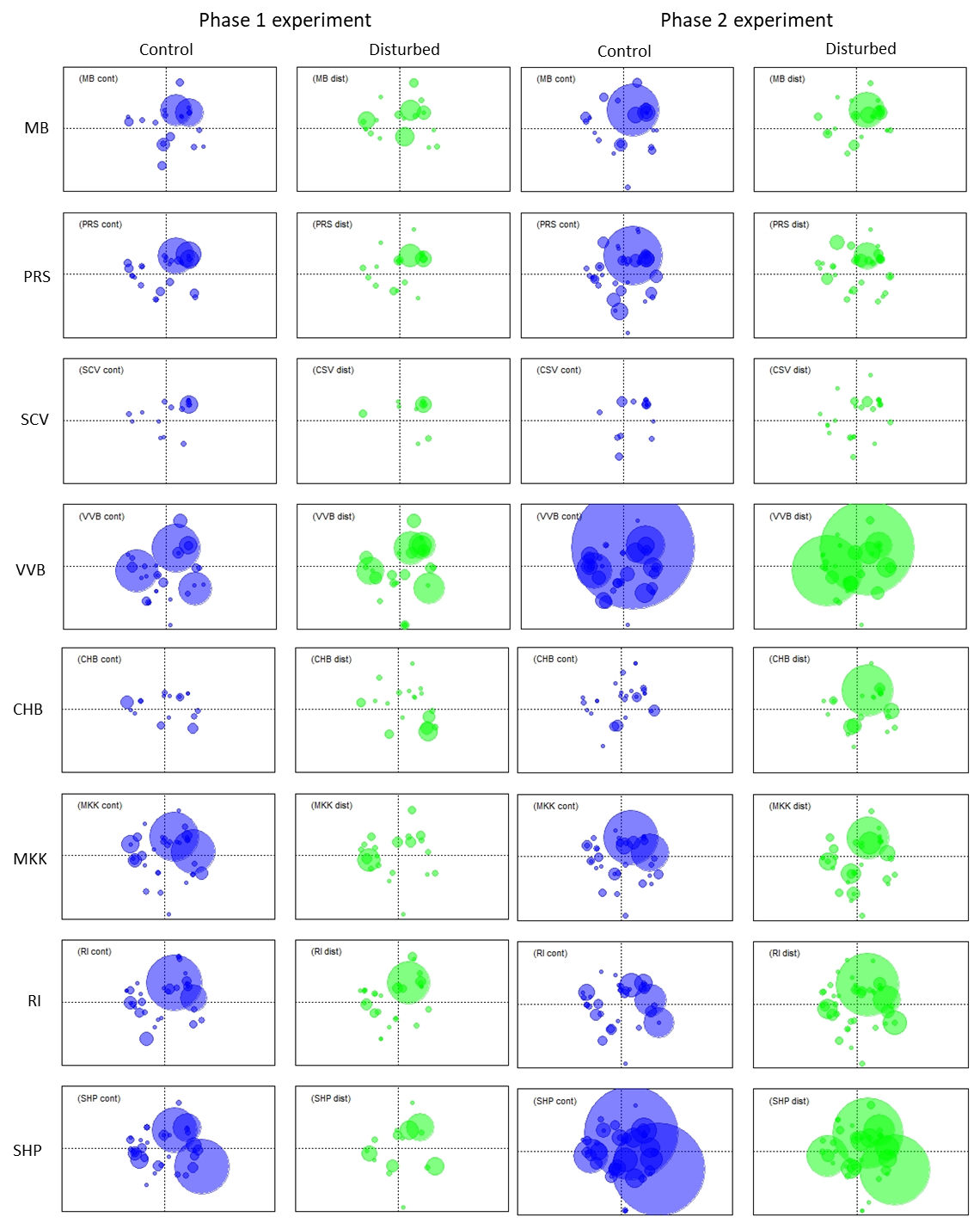


**Fig. A2.3**: PCO ordinations of the community at each site for control (blue) and disturbed (green) plots 5 months after disturbance at Kawau. The plots show the taxa’s position in the individual recovery trait space on axis 1 and 2, and the size of the bubble indicates the abundance of the taxa (sum of three cores at each site).


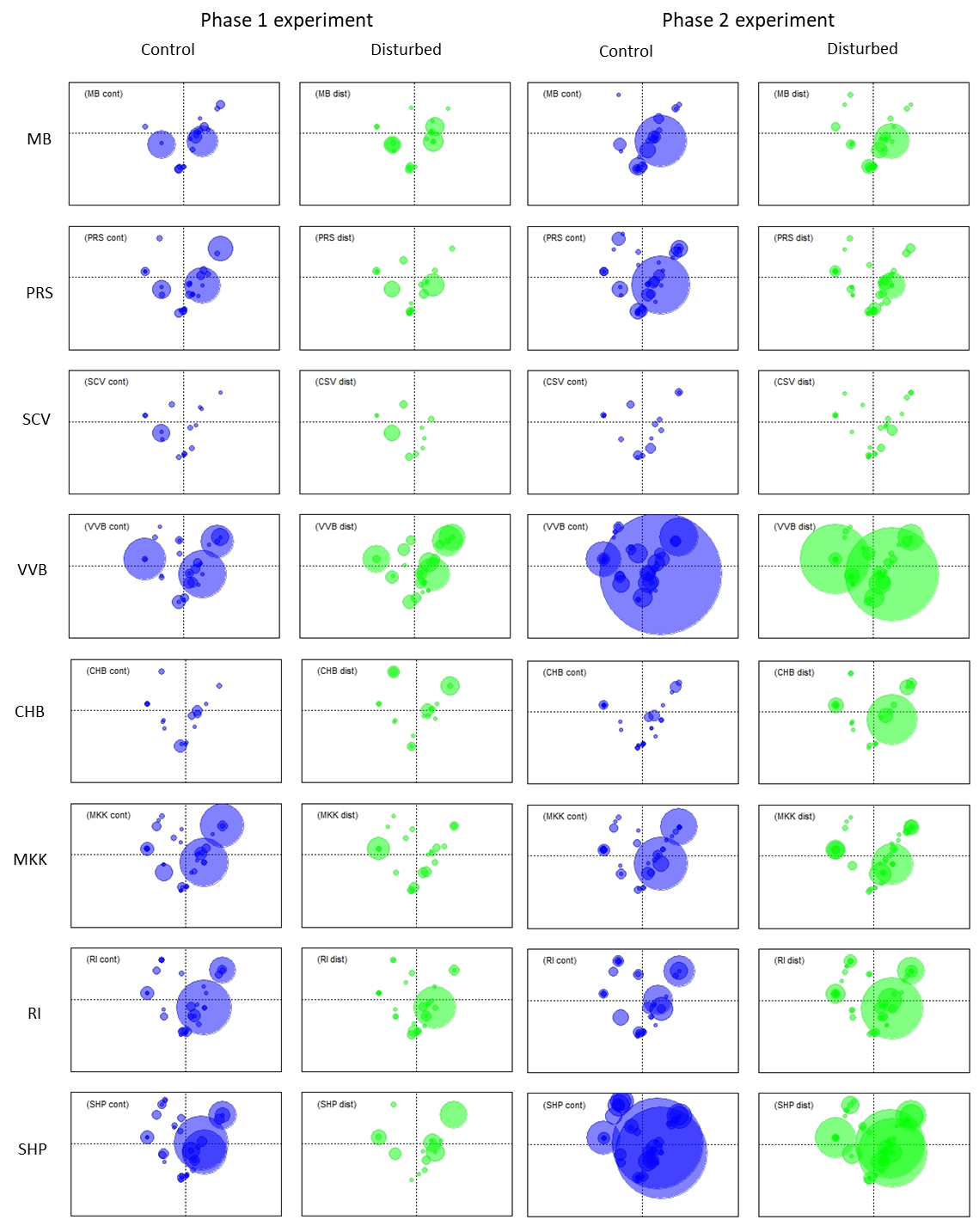


**Fig. A2.4**: PCO ordinations of the community at each site for control (blue) and disturbed (green) plots 5 months after disturbance at Kawau. The plots show the taxa’s position in the landscape recovery trait space on axis 1 and 2, and the size of the bubble indicates the abundance of the taxa (sum of 3 cores at each site).
